# Supplementary material for: Evaluation of Statins Use in Hemodialysis Patients: A Retrospective Analysis of Clinical and Safety Outcomes
Source: Pharmaceuticals (Basel). 2025 Jun 18;18(6):911. doi: 10.3390/ph18060911 (PMC12195794; doi:10.3390/ph18060911)
Supplement: Supplementary file 1 [file pharmaceuticals-18-00911-s001.zip › pharmaceuticals-3639237-supplementary.pdf]

Table S1. Sociodemographic and Clinical Characteristics of Patients Stratified by Individual Statin Types and Non-Statin Therapy:

	Atorvastatin	Rosuvastatin	Simvastatin	Not on Statin Therapy
**Socio-economic and Clinical Variables**	**N (%)**	**N (%)**	**N (%)**	**N (%)**
Male	**41 (33.1)**	**4 (40.0)**	4 (28.6)	15 (48.4)
Female	**83 (66.9)**	**6 (60.0)**	10 (71.4)	16 (51.6)
Age (Mean ± SD)				
Body Mass Index (Mean ± SD)				
**Comorbidities**				
Diabetes Mellitus	103 (83.1)	**7 (70.0)**	12 (85.7)	12 (38.7)
Hypertension	117 (94.4)	**9 (90.0)**	14 (100.0)	28 (90.3)
**CV events**				
None	50 (40.3)	3 (30.0)	7 (50.0)	24 (77.4)
ACS	16 (12.9)	2 (20.0)	0 (0.0)	1 (3.2)
HF	17 (13.7)	2 (20.0)	3 (21.4)	3 (9.7)
ACS and HF	40 (32.3)	3 (30.0)	3 (21.4)	3 (9.7)
RHD with MS	1 (0.8)	0 (0.0)	1 (7.1)	0 (0.0)
**Atherosclerotic Events History**				
None	70 (56.5)	6 (60.0)	10 (71.4)	**22 (71.0)**
Stroke	48 (38.7)	3 (30.0)	4 (28.60)	**9 (29.0)**
PAD	3 (2.4)	0 (0.0)	0 (0.0)	0 (0.0)
Stroke and PAD	3 (2.4)	1 (10.0)	0 (0.0)	0 (0.0)
**Doses**				
10 mg	15 (15.5)	2 (20.0)	3 (21.4)	3 (11.1)
20 mg	39 (40.2)	5 (50.0)	6 (42.9)	6 (22.2)
40 mg	39 (40.2)	3 (30.0)	4 (28.6)	15 (55.6)
60 mg	1 (1.0)	0 (0.0)	0 (0.0)	1 (3.7)
80 mg	3 (3.1)	0 (0.0)	1 (7.1)	2 (7.4)

ACS: Acute coronary syndrome; CV: Cardiovascular events; HF: Heart failure; PAD: Peripheral artery disease; RHD with MS: Rheumatic heart disease with mitral stenosis

Table S1 outlines the baseline characteristics of patients stratified by individual statin type (Atorvastatin, Rosuvastatin, Simvastatin) and those not receiving statin therapy. Across all statin groups, the majority of patients were female, particularly in the Simvastatin group (71.4%). In contrast, the non-statin group had a more balanced gender distribution, with a slightly higher proportion of males (48.4%). Moreover, Patients on statins, particularly Atorvastatin and Simvastatin, had a higher prevalence of diabetes and hypertension compared to non-statin users. Cardiovascular events, including ACS and heart failure, were more frequent among statin users, suggesting they were prescribed to higher-risk individuals. In contrast, non-statin patients had fewer events and a higher proportion with no prior CV history. The most common statin doses were 20 mg and 40 mg, while higher doses were rarely used. Overall, the data reflect a pattern of statin use in patients with more comorbidities and established cardiovascular risk.

Table S2. Mean Survival Times (in Days) to Atherosclerotic Events by Statin Type.

	Mean[a]				Median			
			95% Confidence Interval				95% Confidence Interval	
Statin Types	Estimate	Std. Error	Lower Bound	Upper Bound	Estimate	Std. Error	Lower Bound	Upper Bound
Atorvastatin	642.525	20.647	602.057	682.993	691.000	.000	.	.
Rosuvastatin	395.600	34.532	327.917	463.283	.	.	.	.
Simvastatin	525.600	23.613	479.319	571.881	.	.	.	.
Non-Strains	616.182	32.501	552.481	679.883	669.000	.000	.	.
Overall	626.572	18.913	589.503	663.642	691.000	.000	.	.

[a] Estimation is limited to the largest survival time if it is censored.

Atorvastatin was associated with the longest mean survival time (642.5 days), followed by non-statin users (616.2 days) and Simvastatin users (525.6 days). Rosuvastatin had the shortest mean survival time (395.6 days), although interpretation is limited by its small sample size. Median survival times were not estimable for most groups due to high censoring rates, indicating that the majority of patients remained event-free during the follow-up period.
